# Common causes of vaginal infections and antibiotic susceptibility of aerobic bacterial isolates in women of reproductive age attending at Felegehiwot referral Hospital, Ethiopia: a cross sectional study

**DOI:** 10.1186/s12905-015-0197-y

**Published:** 2015-05-13

**Authors:** Wondemagegn Mulu, Mulat Yimer, Yohannes Zenebe, Bayeh Abera

**Affiliations:** Department of Medical Microbiology, Immunology and Parasitology, College of Medicine and Health Sciences, Bahir Dar University, Bahir Dar, Ethiopia

**Keywords:** BV, Candidiasis, Trichomoniasis, Gonococcal, Women in reproductive age

## Abstract

**Background:**

Bacterial vaginosis, candidal, trichomonal and Gonococcal vaginal infections are a major health problems associated with gynecologic complications and increase in replication, shedding and transmission of HIV and other STIs in women of reproductive age. The study aimed at determining the prevalence of common vaginal infections and antimicrobial susceptibility profiles of aerobic bacterial isolates in women of reproductive age, attending Felegehiwot referral Hospital.

**Methods:**

A hospital based cross sectional study was conducted from May to November, 2013. Simple random sampling technique was used. Demographic variables were collected using a structured questionnaire. Clinical data were collected by physicians. Two vaginal swab specimens were collected from each participant. Wet mount and Gram staining were carried out to identify motile *T.vaginalis*, budding yeast and clue cells. All vaginal specimens were cultured for aerobic bacterial isolates using standard microbiology methods. Antimicrobial susceptibility was performed using disc diffusion technique as per the standard by Kirby-Bauer method. The results were analyzed using descriptive, chi-square and fisher’s exact test as appropriate.

**Results:**

A total of 409 women in reproductive age (15 – 49 years) participated in the study. The median age of the women was 28 years. Overall, 63 (15.4 %) of women had vaginal infections. The proportion of vaginal infection was higher in non-pregnant (17.3 %) than pregnant women (13.3 %) (*P* = 0.002). The most common identified vaginal infections were candidiasis (8.3 %) and bacterial vaginosis (2.8 %) followed by trichomoniasis (2.1 %). The isolation rate of *N. gonorrhoeae* and group B *Streptococcu*s colonization was 4 (1 %) and 6 (1.2 %), respectively. Bacterial vaginosis was higher in non-pregnant (5.6 %) than pregnant women (0.5 %) (*P* = 0.002). Religion, age, living in rural area and having lower abdominal pain were significantly associated with bacterial vaginosis and candidiasis (P < 0.05). *E.coli, Pseudomonas* spp*.* and *S.aureus* were frequently isolated. Norfloxacin (75.6 %), ciprofloxacin (79.6 %) and gentamicin (77.6 %) revealed high level of sensitivity whereas high resistance rates were observed for amoxicillin (82.2 %), tetracycline (63.3 %) and cotrimoxazole (62.2 %).

**Conclusions:**

Bacterial vaginosis, candidiasis and trichomoniasis are a common problem in women of reproductive age. Therefore, screening of vaginal infections in women of reproductive age should be implemented. Moreover, ciprofloxacin, norfloxacin and gentamicin are the recommended drugs for empiric therapy and prophylaxis as needed.

## Background

Vaginal infections with bacterial vaginosis, candidiasis and trichomoniasis are a global health problem for women [1]. Vaginitis is the inflammation and infection of vagina commonly encountered in clinical medicine [2]. Diverse spectrums of pathogenic agents were observed in the vaginal micro flora. Of these, bacterial vaginosis, candidiasis and trichomoniasis are responsible for majority of vaginal infections in women of reproductive age [2, 3].

Abnormal vaginal discharge, itching, burning sensation, irritation and discomfort are frequent complaints among patients attending obstetrics and gynecology clinics. However, a number of vaginal infections present with few or no symptoms [4].

Candida vaginitis (CV) is one of the most frequent infections in women of reproductive age. Approximately 75 % of adult women will have at least one episode of vaginitis by candida during their life time [2, 4]. Unfortunately, about 40 – 50 % of women who had a first episode is likely to present a recurrence and 5 % may present a form of “recurring” characterized by at least three or more episodes of infection per year [5].

Trichomonal vaginitis (TV) is the most common sexually transmitted disease [5]. It is caused by a parasitic protozoan *T. vaginalis* [2]. Globally, TV affects approximately 57–180 million people, with the majority living in developing countries [6]. However, in most cases TV is asymptomatic. In women, TV affects more frequently between 20 and 40 years old and is quite rare before puberty and postmenopausal age [5].

The symptoms of TV are mainly characterized by vaginal discharge with gray or greenish-yellow fluid rather frothy, foul-smelling, intense itching, edema cervix redness, the sensation of itching, dyspareunia and postcoital bleeding, pelvic pain and urinary symptoms [2, 5].

Bacterial vaginosis (BV) is the most common cause of abnormal vaginal discharge among women of reproductive age. The prevalence of BV is about 30 % in women of reproductive age [2, 5]. BV is characterized by raised vaginal pH and milky discharge in which normal vaginal flora (Lactobacilli) is replaced by a mixed flora of aerobic, anaerobic and microaerophilic species. Anaerobic organisms like *Gardnerella vaginalis*, *Prevotella* spp., *Mycoplasma hominis, Mobiluncus* spp. colonize vagina predominantly in BV [5, 7].

Gonococcal infections are the second most common prevalent sexually transmitted bacterial infections causing substantial morbidity worldwide each year. Gonorrhoea is a potent amplifier of the spread of sexually transmitted human immuno deficiency virus (HIV) [8]. Various studies across the world have shown that women with BV are more likely to be co-infected with, *T. vaginalis*, *N.gonorrhoeae* and HIV [7].

Aerobic vaginitis has been identified for a smaller proportion of women whose microbiota (lactobacilli) is dominated by facultative anaerobic or aerobic bacteria especially *S. aureus*, group B streptococci, *E.coli* and *Klebsiella* spp [5, 7].

Vaginal infections are associated with a significant risk of morbidity in women. If untreated they can lead to pelvic inflammatory disease (PID), which can cause long-term sequelae, such as tubal infertility, ectopic pregnancy, reproductive dysfunction and adverse pregnancy outcomes (e.g., preterm labor and delivery and low birth weight). Cervical dysplasia, increased risk of postoperative infection, HIV and Herpes simplex virus (HSV) -1 acquisition and transmission are also resulted from vaginal infections [2, 9–12]. Moreover, BV propagates viral replication and vaginal shedding of the HIV and HSV-2 [11, 12]. Investigators have also reported epidemiologic associations between trichomonas infection and subsequent cervical neoplasia and carcinoma [10].

Various etiologies of vaginal infection results in a number of gynecologic complications and amplify HIV and HSV-1 transmissions. Vaginal infections have neither been the focus of intensive study nor of active control programs in Ethiopia. Therefore, the purpose of this cross-sectional study was to determine the prevalence of common vaginal infections and antimicrobial susceptibility profiles of aerobic bacteria isolates in a reproductive age women attending at antenatal care and gynecology clinics of Felegehiwot referral Hospital, Northwest, Ethiopia.

## Methods

### Study design, period and area

A hospital based cross sectional study was conducted from May to November 2013 at Felege hiwot referral Hospital, Bahir Dar city, Northwest Ethiopia. Felegehiwot referral Hospital is a Regional referral Hospital which has more than 273 beds offering different specialized services. It has an antenatal care and gynecology clinics. The hospital receives referred patients from different areas of the region and provides local emergency services [13].

### Sample size and sampling technique

The sample size was determined using single population proportion formula considering the following assumptions: *P* = 50 % (The expected proportion of reproductive tract infection among women), 95 % confidence level and marginal error of 5 %. Assuming 10 % non-response rate, the sample size was: *n* = 384 + 10 % = 384 + 38 = 422. However, only 409 women in reproductive age completed the questionnaire adequately and provided vaginal swab specimens. Simple random sampling technique was employed to select and include the study participants.

In the study area, more than 15,000 women in reproductive age with or without vaginal discharge get attended in antenatal care and gynecology wards per year and on average 30 patients visited the two clinics per day.

All women in reproductive age attending Felegehiwot referral Hospital were the source population while all women in reproductive age with or without vaginal discharge attending in the antenatal care and gynecology clinics of Felegehiwot referral Hospital were the study population.

### Exclusion criteria

Reproductive age women who have taken antibiotics within seven days at the time of data collection, those with genital malignancy and those who douched their vagina with chemicals were excluded already from the study.

### Variables of the study

#### Dependent variable

Common vaginal infections such as BV, candidiasis and trichomoniasis

#### Independent variables

Demographic variables such as age, religion, residence, marital status, educational and occupational status, pregnancy, history of abortion, oral contraceptive use, use of broad spectrum antibiotics, life time number of sexual partners, presence of lower abdominal pain, abnormal vaginal discharge, vaginal itching and vaginal irritation

### Data collection

Before the actual data collection, questionnaires were pre-tested by taking 43 women of reproductive age attending Felegehiwot referral hospital other than the actual study participant’s. Upon counselling and recruitment, information on demographic variables, pregnancy status, life time number of sexual partners, gestational age, history of abortion and use of oral contraceptive were collected by face to face interview using a structured questionnaire. The clinical diagnosis and clinical data of participants was collected by physicians. Information obtained includes presence of lower abdominal pain, abnormal vaginal discharge, vaginal itching and irritation.

Two vaginal specimens were collected aseptically from study participants using sterile cotton swab with experienced nurses. The swab specimens were immediately dipped into a sterile tube containing two drops of sterile physiological saline and taken to the Microbiology Laboratory of Bahir Dar Regional Health Research and Laboratory Center within five minutes of collection. Direct saline wet mount, Gram staining and bacteriological cultures were carried out for all specimens.

One drop of a vaginal swab suspension with physiological saline were placed on a slide and covered with a cover slip. The wet film were examined under bright field microscopy at 40× objective for the presence of motile trichomonas, pseudohyphae and/or budding yeast cells indicative of candida, granulocytes and clue cells. Gram stained smears were prepared from vaginal swabs and examined under oil immersion at × 1000 magnification to look for clue cells, budding yeasts, granulocytes and Gram negative diplococci.

*Candida* spp was identified by the presence of yeast cells in wet mount as well as identification of > 3 Gram positive budding yeast cells per oil immersion field. *T. vaginalis* was identified by its typical morphology and motility on wet mount of vaginal specimen.

BV was identified by the presence of clue cells which are vaginal epithelial cells with a granular surface and blurred margins because of attached bacteria on a physiological saline (0.85 %) wet mount and presence of Gram negative cocco-bacilli studding vaginal epithelial cells instead of normally predominant Gram positive *Lactobacilli*.

All vaginal specimens were plated on to 5 % sheep blood agar, MacConkey agar, Manitol salt agar, and modified Thayer martin agar to isolate aerobic bacteria. The inoculated media incubated at 37 °C aerobically for 24–72 hours. Modified Thayer martin agar plates incubated in a humidified atmosphere with 5 % carbon dioxide. Identification of the cultured isolate was done by conventional phenotypic and biochemical methods. Gram negative intracellular diplococci which were oxidase and catalase positive were considered as *N.gonorrhoeae.*

Antimicrobial susceptibility testings were performed by Kirby-Bauer disc diffusion method. The following antimicrobial agents were employed: Tetracycline (30 μg), ciprofloxacin (5 μg), norfloxacin (10 μg), gentamycin (10 μg), cotrimoxazole (25 μg), amoxicillin (10 μg), erythromycin (15 μg ) and clindamycin (2 μg ) (Oxoid, England). Resistance data were interpreted according to Clinical and Laboratory Standards Institute (CLSI, 2011). Reference strain of *E.coli* ATCC 25,922 and *S.aureus* ATCC 25,923 were used for quality control for antimicrobial susceptibility test.

### Data analysis

Data was entered and analyzed using Statistical Package for Social Sciences (SPSS) version 20. Descriptive statistics were used to describe the study participants in relation to relevant variables. Chi-square and fisher’s exact test were employed to compare vaginal infections with demographic variables and other factors. *P*-value of < 0.05 was considered statistical significant.

### Ethical clearance

The study was ethically approved by the research and ethical review board of Bahir Dar University. Permission letter was secured from Felegehiwot referral Hospital and written informed consent was obtained from the study participants before proceeding to data collection. Confidentiality of the result was also maintained. Women positive for vaginal infections reported to antenatal care and gynecology clinics gynaecologists for treatment.

## Results

### Demographic characteristics

A total of 409 women in reproductive age with a response rate of 96.9 % participated in the study. The median age of the participants was 28 years (range: 15– 49 years). One hundred ninety five (52.3 %) were pregnant. Majority of participants were asymptomatic to vaginal infections (63.8 %) (Table 1). Majority of participants were urban (71.6 %). Educational levels revealed that 138 (33.7 %) were illiterate and 121 (29.6 %) had education college and above. Three hundred sixty eight (90 %) of participants were orthodox Christian follower and 312 (76.3 %) were married (Table 2). Three hundred ninety (95 %) of participants had 1–3 life time sexual partners and 99 (24.2 %) had previous history of abortion (Table 3).

### Prevalence of vaginal infections

Overall, 63 (15.6 %) women in reproductive age had vaginal infections. The prevalence of vaginal infection was 41 (16.1 %) in asymptomatic women. The prevalence of vaginal infection was higher in non-pregnant 37 (17.3 %) than pregnant women 26 (13.3 %) (*P* = 0.03). The most common identified vaginal infections were candidiasis (8.3 %) and trichomoniasis (2.1 %). However, the most common cause of abnormal vaginal discharge was BV (2.8 %). The most prevalent sexually transmitted infection was *T. vaginalis* 8 (2 %). The proportion of candidiasis was 24 (9.2 %) in asymptomatic women and 20 (9.3 %) in pregnant women. Eleven non pregnant women had BV (5.6 %). However, one pregnant woman had BV (0.5 %). The difference was also significant (*p* = 0.02). BV was noticed in 6 symptomatic women (4.1 %). The proportion of trichomoniasis was 6 (2.3 %) in asymptomatic women and 5 (2.6 %) in non-pregnant women. Group B *Streptococcus* colonization was found in 5 women (1.2 %) where as *N. gonorrhoea* was isolated in 4 women (1 %) of reproductive age. All *N.gonorrhoeae* were isolated from asymptomatic women and all groups B *Streptococcus* were isolated from non-pregnant women (Table 1).

**Table 1:** Frequency of vaginal infections in relation to symptoms and pregnancy in women of reproductive age, Northwest, Ethiopia, 2013

Type of vaginal infections	Asymptomatic women (*N* = 261)	Symptomatic women (*N* = 148)	*P*-value	Pregnant women (*N* = 214)	Non-pregnant women (*N* = 195)	*P*-value	Total
Candidiasis	24 (9.2)	10 (6.8)	0.08	20 (9.3)	14 (7.2)	0.48	34 (8.2)
Bacterial vaginosis	6 (2.3)	6(4.1)	0.73	1 (0.5)	11 (5.6)	0.002	12 (2.9)
Trichomoniasis	6 (2.3)	2 (1.4)	1.00	3 (1.4)	5 (2.6)	0.49	8 (2)
Gonococcus	4 (1.6)	0.0 %	0.58	2 (1)	2 (0.93)	1.00	4 (1)
Group B *Streptococcus*	4(1.5)	1 (0.7)	1.00	0	5 (2.6)	0.02	5 (1.2)
Total	44 (16.9)	19 (12.8)	0.82	26 (13.3)	37 (17.3)	0.03	63 (15.4)

Candidiasis was detected in 14 (13.1 %) women from 30–39 years of age. BV was found in 5 women in the age range of 40–49 years (8.8 %). However, the proportion of trichomoniasis was 5 (2.3 %) in women of age from 20–29 years. Details of vaginal infections with age categories are depicted in Table 2. Candidiasis and trichomoniasis were noticed in 30 (9.6 %) and 8 (25 %) of married women, respectively. Candidiasis was found in 7 (17.1 %) of non- Orthodox religion followers. The difference was also significant (*P* < 0.05). However, all BV cases were detected only from Orthodox religion followers. The proportion of BV was higher in women from rural than urban residents (*P* < 0.05). Moreover, the proportion of BV was higher in women who had farmer educational status than their counter parts (*P* < 0.05). Women who were illiterate and read and write only had nearly 8 (5 %) positivity for BV (Table 2). The proportion of candidiasis was higher in women who had asexual partner for the last 12 months than those had not (*P* < 0.05). BV was detected in 5 women who had history of abortion (6.1 %) and in 6 women who had lower abdominal pain (5.1 %). Overall, more vaginal infections were found in asymptomatic women than women who had different symptoms of vaginitis (Table 3).

**Table 2:** Sociodemographic characteristics of respondents according to distribution of vaginal infection, Northwest, Ethiopia, 2013

Variables	Number of participants	Candidiasis	Bacterial vaginosis	Trichomoniasis
	N (%)	N (%)	N (%)	N (%)
Age of participants				
<20	31 (7.6)	1 (3.2)	1 (3.2)	0
20–29	218 (53.3)	16 (7.3)	5 (2.3)	5 (2.3)
30–39	107 (26.2)	14 (13.1)	1 (0.9)	2 (1.9)
40–49	53 (12.9)	3 (5.6)	5 (9.4)***	1(1.9)
Marital status				
Single	54 (13.2)	2 (3.7)	2 (3.7)	0
Married	312 (76.3)	30 (9.6)	9 (2.9)	8 (25)
Divorced	43 (10.5)	2 (4.7)	1 (2.3)	0
Residence				
Rural	116 (28.4)	8 (6.9)	7 (10.3)***	4 (3.4)
Urban	293 (71.6)	26 (8.9)*	5 (1.7)	4 (1.4)
Religion				
Orthodox	368 (90)	27 (7.3)	12 (2.7)	6 (1.6)
Other	41 (10)	7 (17.1)***	0	2 (0.5)
Educational status				
College and above	121 (29.6)	13 (10.7)	1 (0.8)	0
Highs school	70 (17.1)	9 (12.9)	1 (1.4)	3 (4.3)
Elementary	41 (10)	1 (2.4)	2 (4.9)	0
Read and write	39 (9.5)	4 (10.3)	2 (5.1)	0
Illiterate	138 (33.7)	7 (5.1)	6 (4.3)	5 (3.6)
Occupational status				
Employed	131 (32)	15 (11.5)	1 (0.8)***	1 (0.8)
Farmer	104 (25.4)	9 (8.7)	8 (7.7)	3 (2.9)
Merchant	28 (6.9)	5 (17.9)***	0	1 (3.6)
Students	44 (10.8)	3 (6.8)	2 (4.5)	1 (2.3)
Unemployed	101 (24.8)	2 (2)	1 (1)	2 (2)
Total	409 (100)	34 (8.2)	12 (2.9)	8 (2)

Key: *** *P* < 0.05

**Table 3:** Association of clinical profiles of respondents with vaginal infection, Northwest, Ethiopia, 2013

Characteristics	Number of participants	Candidiasis	Bacterial vaginosis	Trichomoniasis
	*N* (%)	*N* (%)	*N* (%)	*N* (%)
History of abortion				
Yes	99	9 (9.1)	5 (5.1)	1 (1)
No	310	25 (8.1)	7 (2.3)	7 (2.3)
Gestational age				
First trimester	54	4 (7.4)	0	0
Second trimester	61	5 (8.2)	1 (1.6)	1 (1.6)
Third trimester	99	11 (11.1)	0	2 (2)
Oral contraceptive				
Yes	90	7 (7.8)	1 (1.1)	1 (1.1)
No	315	27 (8.6)	11 (3.5)	7 (2.2)
Use of broad spectrum antibiotics				
Yes	107	7 (6.5)	5 (4.7)	0
No	302	27 (8.9)	7 (2.3)	8 (2.6)
Life time number of sexual partners				
1–3	390	33 (8.5)	11 (2.8)	8 (2.1)
≥4	19	1 (5.3)	1 (5.3)	0
Number of sexual partners for the last 12 months				
0	92	5 (5.4)	6 (6.5)***	1 (1.1)
1	310	29 (9.4)***	5 (1.6)	7 (2.3)
≥2	7	0	1 (14.3)	0
Presence of lower abdominal pain				
Yes	122	9 (7.4)	6 (5.1)***	3 (2.5)
No	287	25 (8.7)	6 (2.1)	5 (1.7)
Vaginal douching				
Yes	400	33(8.3)	12(2.9)	8(2)
No	9	1(11.1)	0	0
Vaginal discharge				
Yes	94	8 (8.5)	3 (3.2)	2 (1.1)
No	314	26 (8.3)	8 (2.5)	8 (2.2)
Vaginal itching				
Yes	60	3 (5)	0	0
No	348	31 (8.9)	12 (3.4)	8 (2.3)
Vaginal irritation				
Yes	47	4 (8.5)	1 (2.1)	1 (2.1)
No	361	30 (8.3)	11(3)	7 (1.9)

Key: *** *P* < 0.05

### Antibiotic susceptibility profiles of aerobic bacteria isolates

The most frequent aerobic isolates were *E.coli, pseudomonas* spp and *S.aureus. N. gonorrhoeae* showed 25–50 % resistance rate against ciprofloxacin, tetracycline and erythromycin. Group B *Streptococcus* had 20 % resistance rate against ciprofloxacin, norfloxacin, erythromycin and clindamycin. *E.coli*, *Pseudomonas* spp and *Enterobacter* spp showed high level of susceptibility (60–100 %) for ciprofloxacin, norfloxacin and gentamicin. However, high percentages of resistance were noticed against cotrimoxazole (57.1–80 %), tetracycline (57.1–73.3 %) and amoxicillin (80–85.7 %). *S.aureus* exhibited resistance rates ranging from 67–83 % to tetracycline, amoxicillin and cotrimoxazole. The overall antimicrobial susceptibility demonstrated that ciprofloxacin, gentamicin and norfloxacin revealed high level of sensitivity (75.6–79.6 %). But, 62.2–82.2 % resistance rate was observed in cotrimoxazole, tetracycline and amoxicillin (Table 4).

**Table 4:** Antimicrobial resistance profiles of bacterial isolates from vaginal specimen of women in reproductive age, Northwest, Ethiopia, 2013

Antimicrobial agents	*E. coli*		*S. aureus*		*Pseudomonas* Spp		*Enterobacter* Spp.		*Klebsiella* Spp.		*Proteus* Spp*.*		*Providencia* Spp.		Group B streptococci		*N.gonorrhoeae*		*Total*
	# T	R %	# T	R %	# T	R%	# T	R %	# T	R %	# T	R %	# T	R %	# T	R %	# T	R %	
Ciprofloxacin	15	20	6	33	7	0	5	20	3	33	2	0	2	0	5	20	4	50	10 (20.4)
Norfloxacin	15	20	6	67	7	14.3	5	0	3	33	2	0	2	50	5	20	ND	ND	11 (24.4)
Gentamycin	15	26.7	6	33	7	0	5	40	3	33	2	0	2	0	5	20	4	25	11 (22.4)
Ampicillin	15	73.3	6	83	7	71.4	5	100	3	33	2	0	2	0	5	80	4	25	32 (65.3)
Cotrimoxazole	15	60	6	83	7	57.1	5	80	3	66.7	2	0	2	0	5	80	ND	ND	28 (62.2)
Amoxycillin	15	80	6	83	7	85.7	5	80	3	66.7	2	100	2	100	5	80	ND	ND	37 (82.2)
Tetracyclin	15	73.3	6	67	7	57.1	5	60	3	66.7	2	50	2	50	5	60	4	50	31 (63.3)
Erythromycin	ND	ND	6	50	ND	ND	ND	ND	3	ND	ND	ND	ND	ND	5	20	4	25	5 (33.3)
Clindamycin	ND	ND	6	33	ND	ND	ND	ND	3	ND	ND	ND	ND	ND	5	20	ND	ND	3 (27.3)

# T: number of isolates tested against each antimicrobial agent

R %: percent of isolates resistance to antimicrobial agent, ND: Not done

## Discussion

In present study, the overall prevalence of vaginal infections (15.4 %) was coherent with reports in Kerkuk-Iraq (13.2 %) [14] and India (14.7 %) [15]. However, it was lower compared to reports in Vietnam (49.5 %) [1], Iran (27.6 %) [16] and Thandalam (44 %) [17]. This variation might be methodology difference in isolation and identification of etiologies of vaginal infections. For instance, in this study culture method was not possible to identify BV. Moreover, environmental factors and difference on the actual study participants might also explain the above discrepancy.

In this study, candidiasis followed by BV and trichomoniasis were the leading vaginal infections. This was coherent with a study conducted in Vietnam [1], Bangladesh [18] and Nepal [19] where candidiasis followed by BV was the most prevalent. However, it differs from a study done in India [15] where trichomoniasis was the most prevalent. This study also differs from findings in Shandong [20] where BV was the most prevalent. Comparison was not possible in Ethiopian context owing to lack of reproductive tract infections data in women of reproductive age.

In this study, the prevalence of vaginal infection was significantly higher in non-pregnant compared to pregnant women (*p* = 0.03) which might be due to lowering of immunity in non-pregnant women due to use of steroid drugs as contraceptive. However, significant difference was not found in the proportion of vaginal infections between asymptomatic women and women who had symptoms of vaginitis. This finding was comparable to a study conducted in Nepal [19]. This showed that vaginitis symptoms have multiple etiologies. However, this finding infers the strong recommendation to all women in reproductive age for vaginal infection screening regardless of symptoms of vaginitis and pregnancy status.

The prevalence of candidiasis in the present study (8.3 %) was consistent with reports from kerkuk-Iraq (8 %) [14] and Lebanon (8.8 %) [21]. However, it was higher than a study in India (1.96 %) [15]. In contrast, the prevalence of candidiasis was lower than studies from Vietnam (25.3–34 %), Hanol Vietnam (11.1 %) and Brazil (12.5 %) [1, 22, 23]. This variation could be the difference in study participants as the present study included pregnant, non-pregnant, symptomatic and asymptomatic women in reproductive age.

In this study, prevalence of BV (2.8 %) was in agreement with studies conducted in Hanol Vietnam (3.5 %) and Tribhuvan (2.5 %) [22, 24]. However, it was lower than reports from Addis Ababa, Ethiopia (19.4 %), Tanzania (28.5 %), Brazil (20 %), Iran (26.2 %) and Peru (27 %) [16, 23, 25–27]. This lower prevalence of BV might be associated with the method we used to identify BV. Because, we used only two criteria’s of Amles (wet mount and Gram stain) to identify BV.

The prevailing prevalence of trichomoniasis was comparable to studies carried out in Kerkuk-Iraq (2.9 %), Thandalam (2.1 %), Shandong (2.9 %) and USA (2.8 %) [14, 17, 20, 28]. However, it was higher than a study in Sudan (0.5 %), Vietnam (0.4 %), Turkey (1.1 %) and Hanol Vietnam (1.3 %) [1, 2, 22, 29]. In contrast, the prevalence was lower than studies carried out in Jimma, Ethiopia (4.98 %) [9], Brazil (4.1 %) [23] and India (18.8 %) [30]. The observed difference could be due to variation in pregnancy status, personal hygiene practice, environment, immunity, socioeconomic and cultural factors of the study participants. Moreover, the detection of trichomoniasis by conventional wet mount method in the present study might reduce the actual prevalence.

The distribution of trichomoniasis in the present study was relatively higher among age groups of 20–29 years compared to others. This was comparable to a study done in Kerkuk-Iraq [14]. This might be because this age group constituted the majority of study participants. Moreover, it might be due to the ability of the parasite to alter at the vaginal environment for its survival. On the other hand, peak candidiasis was observed in women of age 30–39 years. This finding conforms to a study finding in Shandong [20]. This is due to the fact that women at this age are more prone to vaginitis related to frequent sexual activities with husbands, pregnancy, weakening of immunity and oral contraceptive use.

In the present study, women aged > 40 years of age had highest proportion of BV. This result conforms to studies conducted in Shandong [20], Indonesia [31] and Bangladesh [32]. It is true that in age ≥ 40 years, the level of estrogen is declining which causes elevated vaginal pH. This condition is not optimal for lactobacilli species growth but very conducive for the growth of microorganisms causing BV.

In this study, all trichomoniasis cases were detected only from married women. This was in line with a study conducted in Nepal [19]. This strengthens the significant role of sexual intercourse in predisposing women to trichomoniasis. Similarly candidiasis was higher in married women compared to others. Similar finding was reported in India [15]. This might be because the married women are more likely to get pregnant and pregnancy is known to be a risk factor for candidiasis. In this study, the highest proportion of BV was noticed among non-pregnant women than pregnant women. This was consistent with reports from Addis Ababa, Ethiopia [25] and Thai women [33]. This could be due to increased coital frequency in non-pregnant compared to pregnant women resulting in reduction in the physiological barrier of the vagina, leads to over growth of normal commensals.

In the present study, all cases of BV and higher proportion of trichomoniasis were detected in women coming from rural area. Moreover, the highest proportion of BV was found in those women with agricultural occupation. This was consistent with a study done in Nepal [19]. The explanation might be because of poor hygiene practices, lack of time to keep their proper health, poor living standard, ignorance and difficulty in accessibility towards immediate health care facilities.

The prevalence of *N. gonorrhoeae* (1 %) in this study was comparable with a study conducted in kerkuk -Iraq (0.8 %), Lebanon (1 %) and Shandong (0.1 %) [14, 20, 27]. Moreover, Group B Streptococc*us* vaginal colonization (1.5 %) in women of reproductive age conforms to a study conducted in South India (2.3 %) [34]. However, it was lower than reports from other part of Ethiopia (20.9 %), Argentina (7.6 %) and Japan [35–37]. This inconsistency might be associated with difference among study participants.

Although, bacterial vaginal infections are one of the major causes of frequent antibiotic use in women of reproductive age, the level of antibiotic resistance in vaginal isolate was not studied before in Ethiopia. Thus, this study presents the antibiogram of the most predominant vaginal isolates. In this study, *E.coli* showed high level of resistance (73–83 %) to tetracycline and amoxycillin and moderate resistance (60 %) to cotrimoxazole. This was consistent with a report in Dessie and Bahir Dar, Ethiopia [38, 39], where 86–90 % and 60–67 % resistance levels of amoxicillin and cotrimoxazole, respectively were noticed. However, in this study *E.coli* and *pseudomonas* spp demonstrated low level of resistance to ciprofloxacin, norfloxacin and gentamycin. This was coherent with reports from Ethiopia and Pakistan [38, 40].

In the present study, *S. aureus* revealed a high level of resistance to amoxicillin. In contrast, *S. aureus* exhibited low levels of resistance (32.4–34.5 %) to ciprofloxacin, gentamycin and norfloxacin. These were consistent with studies from Ethiopia and Pakistan [38, 40].

In the present study, *N.gonorrhoeae* showed 25–50 % resistance rate against ciprofloxacin, tetracycline, ampicillin and erythromycin. This was coherent with studies in Nepal [41], Port Elizabeth [42], and Saudi Arabia [43] where 37.5–42.5 %, 50–94 %, 25 % and 50 % resistance of ciprofloxacin, tetracycline, ampicillin and erythromycin, respectively were noticed. Group B *Streptococcus* revealed 20 % resistance rate against ciprofloxacin, norfloxacin, erythromycin and clindamycin. This was consistent with previous studies in Ethiopia, Uganda and South India [34, 35, 44].

This study was not without limitations thus detecting candidiasis and BV using culture method may be more accurate than microscopic examination but was not possible due to the limitations of laboratory setups. Some of the information was obtained by interview, hence the possibility of recall bias. The study looked at current infection; addition of serology test would have given a broader profile of the infection to include past infection and inability to easily detect some causes of abnormal vaginal discharge due to Chlamydia, *U.urealyticum* and other organisms like viruses by the tools used. Moreover, this is a hospital based study and the occurrence of infection and antibiotic sensitivity may not be representative of the community. However, these data provided comprehensive information on vaginal infections and antimicrobial susceptibility profiles of aerobic bacteria isolates highlight the urgent need for more detailed study in specific group in women of reproductive age using advanced laboratory technique.

## Conclusion

Bacterial vaginosis, candidiasis and trichomoniasis are a common problem in women of reproductive age. Women in rural area, asymptomatic to vaginitis and non-pregnant are the most affected groups. Therefore, screening of vaginal infections in women of reproductive age should be implemented. Moreover, ciprofloxacin, norfloxacin and gentamicin are the recommended drugs for empiric therapy and prophylaxis as needed.

## References

[1] Go VF, Quan VM, Celentano DD, Moulton LH, Zenilman JM (2006). Prevalence and risk factors for reproductive tract infections among women in rural vietnam. Southeast Asian J Trop Med Public Health.

[2] Hacer H, Reyhan B, Sibel Y (2012). To determine of the prevalence of *Bacterial Vaginosis, Candida sp*, mixed infections *(Bacterial Vaginosis + Candida sp), Trichomonas Vaginalis, Actinomyces sp* in Turkish women from Ankara, Turkey. Ginekol Pol.

[3] Spinillo A, Bernuzzi AM, Cevini C, Gulminetti R, Luzi S, Santolo AD (1997). The relationship of bacterial vaginosis, candida and trichomonas infection to symptomatic vaginitis in postmenopausal women attending a vaginitis clinic. Maturitas.

[4] Adeyba OA, Adeoye MO, Adesiji YO (2003). Bacteriological and parasitological vaginitis in pregnant women in iseyin, oyo state, Nigeria. Clin Exp Microbiol.

[5] Prospero FD: Focus on candida, trichomonas, bacteria and atrophic vaginitis. Available at http://womanhealthgate.com/focus-candida-trichomonasbacteria-atrophic-vaginitis/ on July 10, 2014.

[6] Chalechale A, Karimi I (2010). The prevalence of *Trichomonas vaginalis* infection among patients that presented to hospitals in the Kermanshah district of Iran in 2006 and 2007. Turk J Med Sci.

[7] Lamichhane P, Joshi DR, Subedi YP, Thapa R, Acharya GP, Lamsal A (2014). Study on types of vaginitis and association between bacterial vaginosis and urinary tract infection in pregnant women. IJBAR.

[8] Hng NM, Kurtzhals J, Thy TT, Rasch V (2009). Reproductive tract infections in women seeking abortion in Vietnam. BMC Womens Health.

[9] Eshete A, Mekonnen Z, Zeynudin A: *Trichomonas vaginalis* Infection among Pregnant Women in Jimma University Specialized Hospital, Southwest Ethiopia. ISRN Infectious Diseases 2013, 1–5

[10] Saleh AM, Abdalla HS, Satti AB, Babiker SM, Gasim GI, Adam I (2014). Diagnosis of *Trichomonous vaginalis* by microscopy, latex agglutination, diamond’s media, and PCR in symptomatic women, Khartoum, Sudan. Diagn Pathol.

[11] Trabert B (2008). Risk factors for bacterial vaginosis during pregnancy among African-American women. Am J Obstet Gynecol.

[12] Filho DSC, Diniz CG, DASilva VL (2010). Bacterial vaginosis: clinical, epidemiologic and microbiological features. HU Revista Juiz.

[13] Mulu W, Kibru G, Beyene G, Damtie H (2013). Associated risk factors for post operative nosocomial infections among patients admitted at Felegehiwot referral hospital, Bahir Dar, Northwest, Ethiopia. Clin Med Res.

[14] Kadir MA, Sulymaz MA, Dawood IS, Shams- Eldin S (2014). *Trichomonas vaginalis* and associated microorganisms in women with vaginal discharge in Kerkuk-Iraq. Ankara Med J.

[15] Gupta G, Nandwam S, Agarwal A (2013). Prevalence of candidiasis, trichomoniasis and bacterial vaginosis among women of reproductive age group. Indian J Public Health Res Dev.

[16] Bahram A, Hamid B, Zohre T (2009). Prevalence of bacterial vaginosis and impact of genital hygiene practices in non-pregnant women in Zanjan, Iran. Oman Med J.

[17] Mathew R, Sudhakshina R, Kalyani M, Jayakumars S, Lai B, Banu S (2011). Microbiological profile of vaginosis among women of the reprioductive age group, who attended a tertiary care Hospital. JCDR.

[18] Begum A, Nilufar S, Akther K, Rahman A, Khatoon F, Rahman M (2003). Prevalence of selected reproductive tract infections among pregnant women attending an urban maternal and childcare unit in Dhaka, Bangladesh. Health Popul Nutr.

[19] Shrestha S, Tuladhar NR, Basnyat S, Acharya GP, Shrestha P, Kumar P (2011). Prevalence of vaginitis among pregnant women attending Paropakar maternity and women’s Hospital, Thapathali, Kathmandu, Nepal. Nepal Med Coll J.

[20] Xueqiang F, Zhov Y, Yanfang Y, Yutao D, Huiqing L (2007). Prevalence and risk factors of trichomoniasis, bacterial vaginosis, and candidiasis for married women of child-bearing age in rural Shandong. Jpn J Infect Dis.

[21] Ramie S, Kobeissi L, Elkak F, Shamra S, Kreidiel K, Zurayk H (2012). Reproductive tract infections (RTIs) among married non-pregnant women living in a low income suburb of Beirut, Lebanon. JIN Fect Dev Ctries.

[22] An PK, Khanh NT, Ha DT, Chien DT, Thuc PT, Luong PH (2003). Prevalence of lower genital tract infection among women attending maternal and child health and family planning clinics in Hanoi, Vietnam. Southeast Asian J Trop Med Public Health.

[23] Oliveira FA, Pfleger V, Lang K, Heukelbach J, Miralles I, Frage F (2007). Sexually transmitted infections, bacterial vaginosis, and candidiasis in women of reproductive age in rural Northeast Brazil: a population-based study. Mem Inst Oswaldo Cruz Rio Janeiro.

[24] Manandhar R, Sharma J, Pokharel BM, Shrestha B, Pradhan N (2005). Bacterial vaginosis in Tribhuvan University Teaching Hospital. J Inst Med.

[25] Mengistie Z, Woldeamanuel Y, Asrat D, Adera A (2014). Prevalence of bacterial vaginosis among pregnant women attending antenatal care in Tikur Anbessa University Hospital, Addis Ababa, Ethiopia. BMC Res Notes.

[26] Shayo PA, Kihunnwa A, Massinde AN, Mirambo M, Rumanyika RN, Ngwalida N (2012). Prevalence of bacterial vaginosis and associated factors among pregnant women attending at Bugando Medical Centre, Mwanza, Tanzania. Tan J Health Res.

[27] Jones FR, Miller G, Gadea N (2007). Prevalence of bacterial vaginosis among young women in low-income populations of coastal Peru. Int J STD AIDS.

[28] Sutton M, Sternberg M, Koumans EH, McQuillan G, Berman S, Markowitz L (2007). The prevalence of *Trichomonas vaginalis* infection among reproductive-age women in the United States, 2001–2004. CID.

[29] Abdelaziz ZA, Ibrahim ME, Bilal NE, Hamid ME (2014). Vaginal infections among pregnant women at Omdurman Maternity Hospital in Khartoum, Sudan. J Infect Dev Ctries.

[30] Madhivanam P, Bartman MT, Pasutti L, Krupp K, Arun A, Reingold AL (2009). Prevalence of *Trichomonas vaginalis* infection among youth reproductive age women in India: Implications for treatment and prevention. Sex Health.

[31] Ocviyanti D, Rosana Y, Olivia S, Darmawa F (2010). Risk factors for bacterial vaginosis among Indonesian women. Med J Indones.

[32] Yusuf MD, Chowdhury M, Islam KM (2011). Common microbial etiology of abnormal vaginal discharge among sexually active women in Dhaka, Bangladesh. Southeast Asian J Public Health.

[33] Watcharotone W, Sirimai K, Kiriwat O (2004). Prevalence of bacterial vaginosis in Thai women attending the family planning clinic, Siriraj hospital. J Med Assoc Thai.

[34] Sharmila V, Joseph NM, Babu TA, Chaturvedula L, Sistla S (2011). Genital tract group B streptococcal colonization in pregnant women: a South Indian perspective. J Infect Dev Ctries.

[35] Mohammed M, Asrat D, Woldeamanuel Y, Demissie A (2012). Prevalence of group B *streptococcus* colonization among pregnant women attending antenatal clinic of Hawassa health center, Hawassa, Ethiopia. Ethiop J Health Dev.

[36] Quiroga M, Pegels E, Oviedo P, Pereyra E, Vergara M (2008). Antibiotic susceptibility patterns and prevalence of group B S*treptococcus* isolated from pregnant women in misiones, argentina. Braz J Microbiol.

[37] Matasubara K, Nishiyama Y, Katayama K, Yamamoto G, Sugiyama M, Muraj T (2001). Change of antimicrobial susceptibility of group B streptococci over 15 years in Japan. J Antimicrob Chemother.

[38] Abera B, Kibret M (2011). Bacteriology and antimicrobial susceptibility of otitis media at dessie regional health research laboratory, Ethiopia. Ethiop J Health Dev.

[39] Mulu W, Kibru G, Beyene G, Damtie M (2012). Postoperative nosocomial infections and antimicrobial resistance pattern of bacteria isolates among patients admitted at Felege hiwot referral Hospital, Bahir Dar, Ethiopia. Ethiop J Health Sci.

[40] Mumtaz M, Ahmad M, Aftab I, Akhtar N, Hassan M, Hamid A (2008). Aerobic vaginal pathogens and their sensitivity pattern. J Ayub Med Coll Abbottabad.

[41] Bhatta DR, Gokhale S, Ansari MT, Tiwari HK, Gaur A, Mathuria JP (2012). Gonococcal infections: the trends of antimicrobial susceptibility of *Neisseria gonorrhoeae* in Western Nepal. NJMS.

[42] Govender S, Lebani T, Nell R (2006). Antibiotic susceptibility patterns of *Niesseria gonorrhoeae* isolates in Port Elizabeth. S Afr Med J.

[43] Alzahrami AJ, Obeid OE, Hassan MI, Almulhim AA (2010). Screening of pregnant women attending the antenatal care clinic of a tertiary hospital in eastern Saudi Arabia for Chlamydia trachomatis and *Niesseria gonorrhoeae* infections. Indian J Sex Transm Dis AIDS.

[44] Florence AP, Otim F, Okongo F, Ogwang M, Greco O (2012). The prevalence and antibiotics susceptibility pattern of *Neisseria gonorrhoeae* in patients attending OPD clinics at St. Mary’s Hospital Lacor Uganda.. J Prev Med Hyg.

